# Systematic Evaluation of Kinetics and Distribution of Muscle and Lymph Node Activation Measured by ^18^F-FDG- and ^11^C-PBR28-PET/CT Imaging, and Whole Blood and Muscle Transcriptomics After Immunization of Healthy Humans With Adjuvanted and Unadjuvanted Vaccines

**DOI:** 10.3389/fimmu.2020.613496

**Published:** 2021-02-05

**Authors:** Zarni Win, January Weiner 3rd, Allan Listanco, Neva Patel, Rohini Sharma, Aldona Greenwood, Jeroen Maertzdorf, Hans-Joachim Mollenkopf, Kat Pizzoferro, Thomas Cole, Caroline L. Bodinham, Stefan H. E. Kaufmann, Philippe Denoel, Giuseppe Del Giudice, David J. M. Lewis

**Affiliations:** ^1^ Department of Nuclear Medicine and Radiological Sciences Unit, Imperial College Healthcare NHS Trust (ICHNT), London, United Kingdom; ^2^ Department for Immunology, Max Planck Institute for Infection Biology, Berlin, Germany; ^3^ Core Unit for Bioinformatics (CUBI), Berlin Institute of Health, Berlin, Germany; ^4^ National Institute for Health Research (NIHR) Imperial Clinical Research Facility (NICRF), Imperial College Healthcare NHS Trust, London, United Kingdom; ^5^ Department of Surgery & Cancer, Imperial College London (ICL), London, United Kingdom; ^6^ Surrey Clinical Research Centre, University of Surrey, Guildford, United Kingdom; ^7^ External R&D, GSK, Rixenstart, Belgium and Siena, Italy

**Keywords:** PET/CT (positron emission tomography/computed tomography), transcriptomics, systems vaccinology, reactogenicity, muscle, fluorodeoxyglucose (F-FDG) 18, TSPO (18kda translocator protein)

## Abstract

Systems vaccinology has been applied to detect signatures of human vaccine induced immunity but its ability, together with high definition *in vivo* clinical imaging is not established to predict vaccine reactogenicity. Within two European Commission funded high impact programs, BIOVACSAFE and ADITEC, we applied high resolution positron emission tomography/computed tomography (PET/CT) scanning using tissue-specific and non-specific radioligands together with transcriptomic analysis of muscle biopsies in a clinical model systematically and prospectively comparing vaccine-induced immune/inflammatory responses. 109 male participants received a single immunization with licensed preparations of either AS04-adjuvanted hepatitis B virus vaccine (AHBVV); MF59C-adjuvanted (ATIV) or unadjuvanted seasonal trivalent influenza vaccine (STIV); or alum-OMV-meningococcal B protein vaccine (4CMenB), followed by a PET/CT scan (n = 54) or an injection site muscle biopsy (n = 45). Characteristic kinetics was observed with a localized intramuscular focus associated with increased tissue glycolysis at the site of immunization detected by ^18^F-fluorodeoxyglucose (FDG) PET/CT, peaking after 1–3 days and strongest and most prolonged after 4CMenB, which correlated with clinical experience. Draining lymph node activation peaked between days 3–5 and was most prominent after ATIV. Well defined uptake of the immune cell-binding radioligand ^11^C-PBR28 was observed in muscle lesions and draining lymph nodes. Kinetics of muscle gene expression module upregulation reflected those seen previously in preclinical models with a very early (~6hrs) upregulation of monocyte-, TLR- and cytokine/chemokine-associated modules after AHBVV, in contrast to a response on day 3 after ATIV, which was bracketed by whole blood responses on day 1 as antigen presenting, inflammatory and innate immune cells trafficked to the site of immunization, and on day 5 associated with activated CD4+ T cells. These observations confirm the use of PET/CT, including potentially tissue-, cell-, or cytokine/chemokine-specific radioligands, is a safe and ethical quantitative technique to compare candidate vaccine formulations and could be safely combined with biopsy to guide efficient collection of samples for integrated whole blood and tissue systems vaccinology in small-scale but intensive human clinical models of immunization and to accelerate clinical development and optimisation of vaccine candidates, adjuvants, and formulations.

## Introduction

While licensed vaccines are generally safe and effective, their development is both time consuming and expensive requiring many thousands of trial participants to generate a safety database before licensing, and potentially ongoing surveillance thereafter. This can lead to difficult decisions about selection of dose, formulation, schedule and other factors (1) that are rarely fully explored during development but may lead to failure in Phase 3 efficacy trials, or unexpected safety concerns post licensing (2–4). While such failures are unwelcome at any time they are especially acute during epidemics or pandemics, such as recent outbreaks of Ebola, SARS-CoV-1 and SARS- CoV-2 where vaccine development may be expedited. The potential for small-scale but intensive clinical studies that may identify biomarkers of both vaccine efficacy and safety/reactogenicity is therefore attractive (5). While the use of whole blood transcriptomics profiling (“systems vaccinology”) is well established for identification of biomarkers of immunogenicity (6, 7), very little has been established to predict vaccine safety or reactogenicity. With increasing use of adjuvants, live viral or other novel delivery systems such as RNA, the regulatory field is increasingly exploring the application of high throughput, precision techniques in early phase clinical trials to augment pre-clinical models for the evaluation and optimisation of candidate vaccine formulations (8). With this in mind the European Commission supported two High Impact research programs: “ADITEC” for the application of advanced immunology techniques for more effective vaccines (9, 10); and *via* the Innovative Medicines Initiative-Joint Undertaking (11), “BIOVACSAFE” to assess the ability of systems vaccinology and other high throughput, precision technologies to develop clinical and pre-clinical biomarkers of vaccine safety and reactogenicity (12).

Increased muscle and lymph node ^18^F-FDG PET/CT activity following immunization has been described in scattered case reports (13–16), or in retrospective series of patients with oncological or inflammatory conditions (17–20) serendipitously scanned sometime after immunization. Lymph node activity was generally highest within 7–12 days, e.g., after H1N1 influenza vaccines (19) especially those adjuvanted with AS03 (DL-α-tocopherol (vitamin E), squalene and polysorbate 80) or MF59C (17, 18). Similarly in a small study of healthy female trial volunteers receiving either alum- or AS04-adjuvanted Human Papilloma Virus vaccines (21) axillary lymph node activity was universally seen sometime between 8 and 14 days after immunization, with decreased frequency after 30 days, and contralateral activity only after AS04 adjuvanted vaccine. However these studies were generally opportunistic and the participants generally unrepresentative of early stage vaccine trials. We therefore prospectively and systematically characterized early tissue responses to unadjuvanted and adjuvanted vaccines in healthy adult volunteers, with a special focus on the intramuscular site of immunization as a marker of vaccine reactogenicity.

We have already reported the application of systems vaccinology in BIOVACSAFE to identify biomarkers of reactogenicity in preclinical models (22); and using whole blood in small but highly intensive comparative clinical studies of vaccines and adjuvants (23), and in pregnancy (24). We report here linked clinical studies applying precision positron emission tomography/computed tomography (PET/CT) scanning with tissue-specific and non-specific radioligands, and systems vaccinology analysis of gene expression changes in local tissue at the site of immunization, to systematically and prospectively compare inflammatory and immune responses to a range of adjuvanted and unadjuvanted licensed vaccines (**Table 1**). These studies provide a safe and ethical clinical model that can be applied to the evaluation and optimisation of novel vaccine candidates, adjuvants, and formulations in early phase clinical development.

**Table 1:** Summary of vaccines, antigens, adjuvants and principal findings on PET/CT and Gene Expression.

Vaccine acronym	Antigen/manufacturer	Adjuvant	Principal findings	
			PET/CT	Gene expression
**4CMenB**	Meningococcal group B subunit proteins, Outer Membrane Vesicles/GSK	alum	↑↑↑ muscle, peak D5, remains ↑ ↑ lymph nodes, peak D5	
**AHBVV**	Hepatitis B virus surface antigen/GSK	AS04C: 3­*O*­desacyl­4’­ monophosphoryl lipid A (MPL) adsorbed on aluminium salt		**Blood**: ↑D3: innate immunity, IFNs
				**Muscle**: ↑↑3h: monocytes, TLR signalling, antigen presentation, cytokines, neutrophils
**ATIV**	Subunit seasonal trivalent influenza vaccine/Seqirus Vaccines and Diagnostics	MF59C: squalene, polysorbate 80, sorbitan trioleate, oil-in-water emulsion	↑↑ muscle, peak D3 then ↓ ↑↑lymph nodes, peak D5	**Blood**: ↑↑D1: innate immunity, IFNs, dendritic cells, neutrophils, monocytes ↑↑D5: CD4 T cells
				**Muscle**: ↑↑↑D3: antigen presentation, monocytes, TLR signalling, cytokines, IFNs, dendritic cells, T cells
**STIV**	Split virion inactivated seasonal trivalent influenza vaccine/Sanofi Pasteur		↑ muscle, peak D3 then ↓ ↑ lymph nodes, peak D3	

## Materials and Methods

### Participants, Immunizations, Muscle Biopsy and Reactogenicity Scoring

#### ^18^F-FDG- and ^11^C-PBR28 -PET/CT Imaging Protocol

Male participants aged 18–55 deemed healthy by medical history and symptoms-directed physical examination were eligible for enrolment at the NICRF, London subject to the following inclusion criteria: able to understand and signed the informed consent form (ICF); body mass index 19–27 kg/m^2^; pre-immunized with a hepatitis B vaccine on the basis of immunization history if AHBVV to be the study vaccine, and have not received any meningococcal B vaccine on the basis of immunization history if 4CMenB to be the study vaccine (immunization history and existing immunity not recorded for other vaccines); have not undergone research radiation exposures, and agree to avoid such exposures for 12 months before/after this study; willing to avoid vigorous exercise or contact sports between vaccination and PET scan; no history of hypersensitivity to any of the vaccine components or a history of any allergy; no use of steroids or immunosuppressive/immunomodulating drugs either orally or parenterally within 3 months of the PET scan; not currently participating in a clinical study with a drug or device; do not express TSPO with low-affinity to PBR28 on the basis of TSPO genotype (^11^C-PBR28 PET imaging only).

All immunizations were a 0.5 ml intramuscular injection on one occasion, randomised to right or left side, using a 25 mm long 23 gauge (G) needle into the antero-lateral thigh with the manufacturer recommended dose. In the ^18^F-FDG PET/CT protocol participants received one of the following commercially sourced vaccines: 4CMenB (Meningococcal group B subunit/Outer Membrane Vesicles - alum, GSK); AHBVV (Hepatitis B virus surface antigen adjuvanted by AS04C containing 3­*O*­desacyl­4’­ monophosphoryl lipid A (MPL) adsorbed on aluminium salt, GSK); ATIV (subunit seasonal trivalent influenza vaccine adjuvanted with MF59C, Seqirus Vaccines and Diagnostics) or STIV (split virion inactivated seasonal trivalent influenza vaccine, Sanofi Pasteur). Those receiving STIV were also injected with saline control in the contralateral leg. In the ^11^C-PBR28-PET protocol participants received 4CMenB and saline control in the contralateral leg.

^18^F-FDG PET/CT imaging participants completed a daily diary card recording and scoring severity of solicited symptoms of redness or swelling (score 0 or 1); injection site pain, feeling hot, headache, myalgia, arthralgia, malaise, nausea/vomiting, and an overall score (score 0–4 each). Individual symptom scores were added together to calculate a total reactogenicity score for each subject on each day after immunization.

#### Muscle Biopsy Transcriptomics Protocol

Male participants aged 18–55 deemed healthy by medical history and symptoms-directed physical examination were eligible for enrolment at the Surrey Clinical Research Centre, Guildford subject to the following inclusion criteria: able to understand and signed the ICF; body mass index 19–27 kg/m^2^; pre-immunized with a hepatitis B virus (HBV) vaccine on the basis of immunization history and seropositive for anti-HBV sAb, negative for HBV sAg and cAb (evidence of HBV immunization but not prior infection; ATIV immunization history and existing immunity not recorded); human immunodeficiency virus 1 and 2 antibodies negative; hepatitis C virus antibodies negative; no history of hypersensitivity to any of the vaccine components, injected local anesthetics or a history of any allergy; no use of steroids or immunosuppressive/immunomodulating drugs either orally or parenterally within 3 months of the immunization; not currently participating in a clinical study with a drug or device; no known immune or coagulation disorder or clinically significant abnormalities of platelets, hemoglobin or coagulation on screening blood tests.

On the day of immunization blood was first drawn into PaxGene tubes for whole blood transcriptomics. Participants then received a 0.5 ml intramuscular immunization, randomised to right or left side, using a 25 mm long 23G needle introduced at right angles to the full length of the needle into the antero-lateral thigh (targeting vastus lateralis muscle) with the manufacturer recommended dose of one of the following commercially sourced vaccines: AHBVV, ATIV or normal saline control. The injection site was marked with indelible marker.

On the allocated day of muscle biopsy blood was first drawn into PaxGene tubes for whole blood transcriptomics. The biopsy was obtained using a well-established technique (25): the site of immunization, and an anatomically matched site on the unimmunized contralateral leg was identified and sterilized using 4% w/v chlorhexidine before being infiltrated intradermally and subcutaneously with 2–5 ml 1% lidocaine (without epinephrine) using a 23G needle. After approximately 5 min a 21G needle was advanced slowly at a 45 degree angle into the subcutaneous tissue, taking care not to penetrate the muscle fascia, and the biopsy site infiltrated with 10–15 ml 1% lidocaine (without epinephrine) in a diamond pattern. This technique ensured that the subcutaneous tissue and muscle fascia is anesthetized without affecting the muscle sample to be biopsied. After a further 5 min a 5–10 mm stab incision was made with a surgical blade inserted far enough to make a small incision in the muscle fascia. A modified Bergström needle with a Luer lock attachment to the inner cannula to allow application of suction during the procedure, and with the exact depth of the needle used for immunization engraved on the shaft, was used to obtain the biopsy. The needle was introduced perpendicularly, through the muscle fascia to the marked depth and a slight vacuum created by pulling back on the plunger of an attached 60 ml syringe. Simultaneously the hole at the top of the Bergström needle was sealed and the inner trocar with the cutting edge pulled up, opening the cutting window in the outer needle. The trocar was then pushed down to excise a piece of muscle and repeated three to four times while maintaining the vacuum. The needle was withdrawn, the inner cannula removed and the muscle sample removed with non-toothed forceps or hypodermic needle tip and immediately placed in 1.5 ml RNA*later* (Invitrogen) reagent in a 2 ml tube. After at least overnight storage at 4°C the tubes were transferred to -80°C freezer for storage prior to RNA extraction and analysis in batches.

### PET/CT Scanning and Image Analysis

In order to keep the radiation dose below acceptable exposure levels of 10 milliSieverts (mSv) per annum in this healthy young adult population, each participant had a single PET/CT scan on one occasion only. To calibrate the ^18^F-FDG PET/CT scan an iterative time series was initially performed at the Clinical Imaging Facility (CIF), Imperial College over 20–60 min after ^18^F-FDG administration to one participant who had received AHBVV three days previously. Uptake in the muscle ROI increased until it reached a plateau between 50–60 min (data not shown). Therefore, a standard ^18^F-FDG PET/CT scan protocol was employed thereafter as follows: the participants’ height and weight were recorded and plasma glucose checked before a weight corrected dose of ^18^F-FDG (2.9 MBecquerel/kg [MBq/kg] with a dose constraint of 200 MBq, effective dose 4.0 mSv) was injected intravenously. The participant then remained resting to avoid any muscle use until 55 min post injection when they voided the urinary bladder before being placed in a Siemens Biograph 64 scanner. A CT topogram for position was performed followed by an attenuation correction two bed positions (bp) low dose CT scan from the pelvic brim down to just past vaccine injection site (120 keV, 100mA, rotation 0.5 s, 50 mAs, total CT dose restricted to 2.5 mSv). This was followed by a two bp static PET scan linked to the first CT, then a half body non-attenuation corrected PET scan from eyes to mid-thigh. This protocol was confirmed by scanning a second participant at the CIF 3 days after immunization with AHBVV, and used thereafter for the other study vaccines for which scans were performed at the Department of Nuclear Medicine and Radiological Sciences Unit, Imperial College Healthcare NHS Trust.

For analysis, ^18^F-FDG -PET/CT uptake was converted into dose- and weight-corrected Standardised Uptake Values (SUV) (26) and attenuation corrected and reconstructed PET images created using the CT scan and Ordered Subset Expectation Maximization (OSEM) algorithms with 4 iterations and 8 subsets, and the Siemens scanner software. DICOM images were imported into Osirix MD (Pixmeo, Geneva. FDA cleared as a Class II Medical Device for diagnostic imaging in medicine, compliant with European Directive 93/42/EEC concerning medical devices, Class IIa product) for calculation of regions of interest (ROI) of increased activity at muscle injection site and lymph nodes using inbuilt minimum-maximum thresholding algorithms. Standard parameters for PET quantification (26) were calculated using the Osirix software tools: SUVpeak (average SUV of a 1 cm^3^ sphere centered on the SUVmax voxel); Volume Of Interest (VOI, by combining and interpolating 2D ROI) and SUVmax, SUVmean and Total Lesion Glycolysis (SUVmean x VOI cm^3^) within the 3D VOI. A fixed threshold of 0.9 SUV was found to give optimal muscle segmentation at the different time points after immunization. In addition an Osirix software plugin (available at https://github.com/djmlewis/mirrorroi.git) was created which used anatomical features on the CT scan to accurately mirror each 2D ROI onto the contralateral uninjected leg for calculation of control SUVmax and SUVmean. Scans were analyzed by an experienced consultant radiology and nuclear medicine clinician (ZW) blinded to treatment, who identified increased lymph node activity and anatomical location.

Individual ^11^C-PBR28 doses were prepared using an on-site cyclotron and used immediately. For the ^11^C-PBR28 PET/CT protocol, a weight adjusted ^11^C-PBR28 (target activity 400 MBq, effective dose 2.64 mSv) PET/low dose CT scan (1.26 mSv dose constraint) was performed at Invicro, London on days 1, 3, 5, or 7 after immunization. Preliminary time-series analysis revealed a rapid accumulation of ^11^C-PBR28 at injection site over 15 min with a plateau thereafter to 80 min (data not shown). Thereafter, ^11^C-PBR28 analysis was performed on acquisition between 30–50 min after ^11^C-PBR28 administration using OSEM algorithms with 3 iterations and 21 subsets.

### Whole Blood and Muscle Biopsy Transcriptomics Analysis

#### Microarray Analysis, Normalization and Quality Control

RNA was extracted from whole blood in PaxGene tubes (PreAnalytiX) on the automated QIAcube system (Qiagen) using the PaxGene Blood RNA kit (Qiagen) according to the manufacturer’s instructions. Muscle biopsies in RNA*easy* were defrosted before total RNA isolation (including microRNA [miRNA] species) was performed using the miRNeasy mini kit (Qiagen, UK), as described previously (22). Quality control and quantification of isolated RNA was analyzed with an Agilent 2100 Bioanalyzer (Agilent Technologies) and a NanoDrop 1000 UV-Vis spectrophotometer (Thermo Fisher Scientific). Microarray experiments were performed as single-color hybridization, and RNA was labeled with the Low Input Quick-Amp Labeling Kit (Agilent Technologies) as described previously (23). Scanning of microarrays was performed with 3 μm resolution and 20-bit image depth, using a G2565CA high-resolution laser microarray scanner (Agilent Technologies). Microarray image data were processed with the Image Analysis/Feature Extraction software G2567AA v. A.11.5.1.1 (Agilent Technologies), using default settings and the GE1_1105_Oct12 extraction protocol. Blinded primary readouts of the microarrays were read, background corrected, normalized and controlled for quality using the R package limma (23). The normalized data were locked and unblinded for further analysis. All primary readouts and the background corrected and normalized data are available from the Gene Expression Omnibus database (GEO, accession number GSE124719) and under the BioProject identifier PRJNA513261.

#### Differential Gene Expression, Gene Set Enrichment, and Individual Variability Analysis

Prior to analysis, hybridization control samples were removed from the data set, and gene expression values averaged for each probe over all replicates of that probe on the microarray, using the ‘avereps’ function from limma (23). Differences in gene expression for each vaccine at each time point tested were assessed using linear models in limma and p-values corrected for false discovery rate using the Benjamini and Hochberg (BH) procedure (23). For whole blood, the expression was fit to time point, vaccine group and subject and the contrast tested for a given vaccine and a given time point was the interaction between the difference in expression between this time point and the pre-immunization time point. For muscle biopsy, the difference in expression was between the biopsy from the injection site and the biopsy from the uninjected leg; or the vaccine group and the saline control injection group for the injected leg biopsy only. Gene set enrichment was tested with the CERNO algorithm implemented in the R package tmod (23, 27). Area under curve (AUC) as described previously (27) was used as effect size estimate. P-values from the CERNO test were corrected using the Benjamini and Hochberg (BH) procedure. For testing, the gene sets [blood transcriptional modules (BTMs)] defined by Li et al (28). and Chaussabel et al (29). were used. p-values were corrected using the BH procedure; gene set enrichments with p_adjusted_ < 0.05 were considered significant. Individual variability was analyzed by ordering genes according to a difference in expression between the relevant condition and a comparable control and applying the CERNO algorithm and the above mentioned gene sets to the list of genes ordered by decreasing absolute difference. For blood, the difference in expression between the given time point and the baseline was calculated and used to order the genes. For biopsies, the difference between the uninjected site and the vaccine injection site was used to order the genes.

### Ethics Approval and Registry

The PET/CT imaging and muscle transcriptomics protocols were approved by the London–Surrey Borders Research Ethics Committee (references 15/LO/2039 and 14/LO/2226 respectively) and registered on clinicalTrials.gov (NCT02368327 and NCT04515368). The administration of radionucleotides and the PET/CT scanning protocol were approved by the UK Administration of Radioactive Substances Advisory Committee (research certificate number RPC 44/3681/35747).

## Results

### Participants and Immunizations

A total of 109 male participants were enrolled sequentially into the treatment groups at two study sites. For ^18^F-FDG PET/CT, 54 participants (median age 28, range 19–55 years) were enrolled and immunized in the NICRF with either 4CMenB (n = 21), ATIV (n = 19), STIV (n = 12, and with simultaneous saline control in contralateral leg) or AHBVV (n = 2). ^18^F-FDG PET/CT scans were performed in the Clinical Imaging Facility, Imperial College London on day 3 after AHBVV to define the scanning protocol. Thereafter, ^18^F-FDG PET/CT scans were performed in the Department of Nuclear Medicine, Imperial College Healthcare NHS Trust at 3 hours, 1, 3, 5, 7, or 10 days after immunization with 4CMenB and ATIV; and at 3 hours, 1, 3, and 7 days after STIV. For ^11^C-PBR28-PET/CT, 10 participants (median age 25, range 21–51 years) were enrolled and immunized in the NICRF with 4CMenB (and with simultaneous saline control in contralateral leg) and scans performed 1, 3, 5, or 7 days after immunization at Invicro, London. For whole blood and muscle biopsy transcriptomics, a total of 45 male participants (median age of 18, range 23–36 years) were enrolled, immunized with either AHBVV, ATIV or saline control (n = 15 per treatment group, n = 3 per time point per group) and biopsied in the Surrey Clinical Research Centre at 3 hours, 1, 3, or 5 days post-immunization.

### ^18^F-FDG and ^11^C-PBR28 PET Activity in Muscle at Site of Immunization

**Figure 1:**
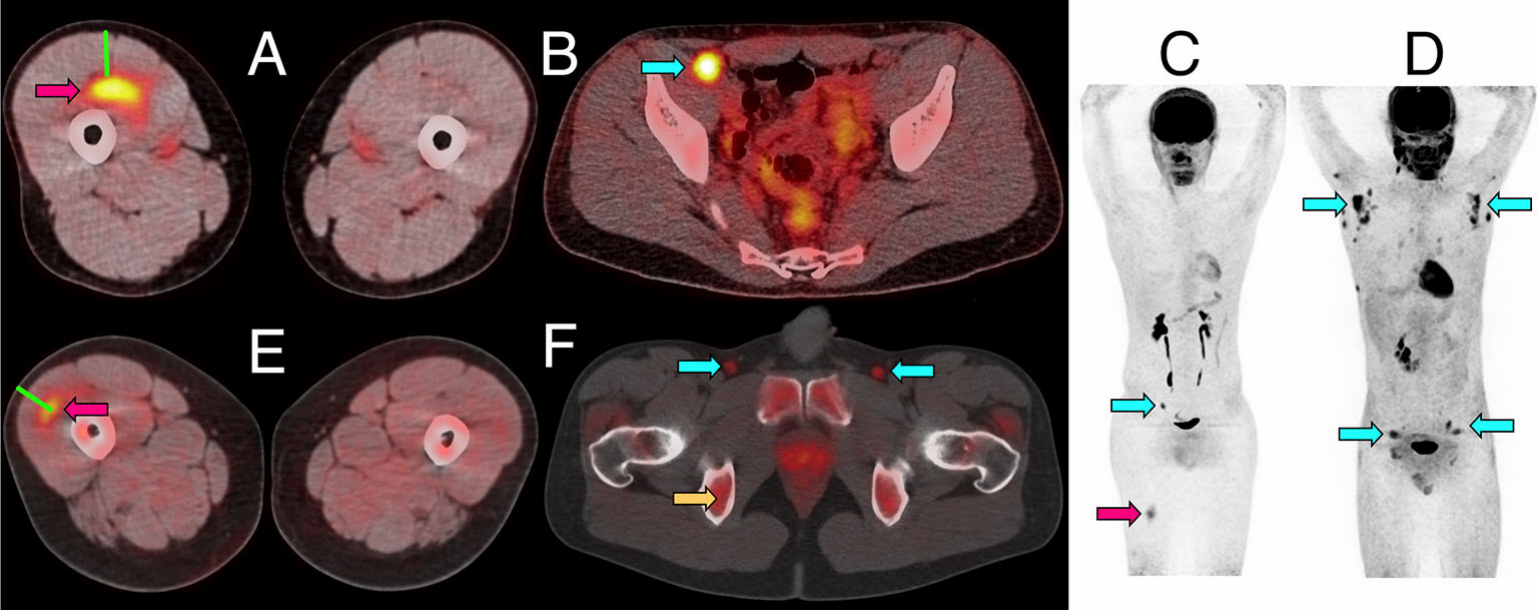
Representative PET/CT images of intramuscular injection site and draining lymph nodes. **(A**–**C)** Fused ^18^F-FDG PET/CT images (linear scale) from one participant 5 days after immunization with 4CMenB, demonstrating increased activity at right anterior thigh intramuscular injection site (**A**, red arrow) and right external iliac lymph node (**B**, cyan arrow****); whole body PET scan anterior view **(C)** demonstrating level of injection site (red arrow) and activated lymph node (cyan arrow). **(D)** Whole body ^18^F-FDG PET scan anterior view demonstrating disseminated axillary and pelvic lymph node activity (cyan arrows) 1 day after immunization with ATIV. **(E**, **F)** Fused ^11^C-PBR28-PET/CT images (linear scale) from two participants immunized with 4CMenB demonstrating increased ^11^C-PBR28 binding in right thigh injection site (**E**: red arrow, day +3****); and right and left deep inguinal lymph nodes (**F**: cyan arrows, day +7, right thigh injected). Increased bone marrow binding of ^11^C-PBR28 also visible (**F**: yellow arrow****). Green bars **(A**, **E)** indicate length of immunization needle, actual track unknown. Diffuse uptake of ^18^F-FDG can be seen in bowel **(B)**, and for both radionucleotides in urinary collecting system and bladder **(B–D, F)**.

**Figure 2:**
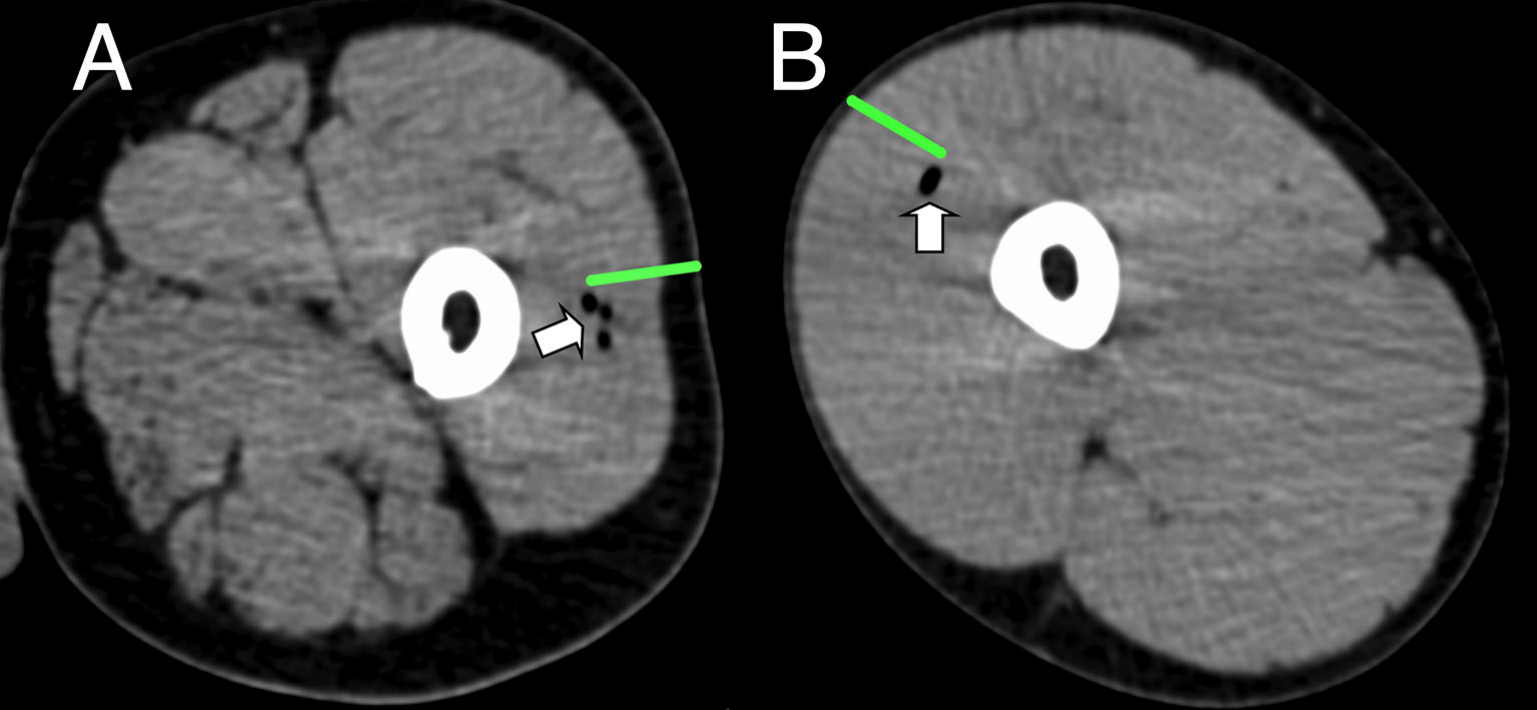
Representative CT imaging of intramuscular immunization site 3 h post injection. Transient inclusions (white arrows) visible at site of immunization with ATIV **(A)** and 4CMenB **(B)**. Green bars indicate length of immunization needle, actual injection track unknown.

**Figure 3:**
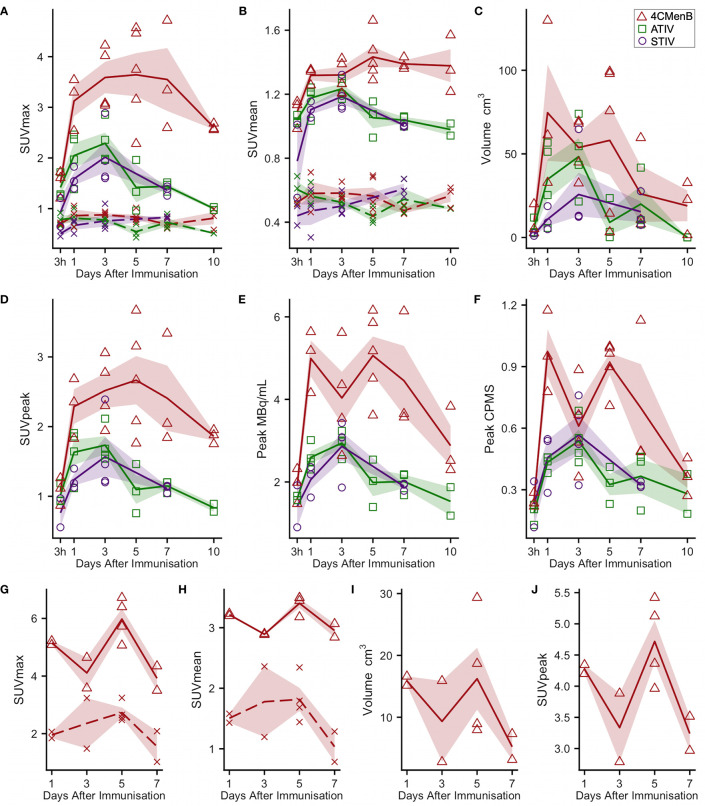
Kinetics of injection site muscle PET/CT activity after immunization **(A**–**F)** ^18^F-FDG PET/CT. Panels **(G–J)**: ^11^C-PBR28 PET/CT. Each point indicates data from one participant immunized with 4CMenB (red triangles), ATIV (green squares) or STIV (purple circles). Lines indicate group mean by vaccine and day of scan, shaded area: SEM. For SUVmax and SUVmean: solid lines: immunized muscle; broken lines/crosses: contralateral muscle.

^18^F-FDG PET/CT indirectly detects metabolically hyperactive cells by quantifying the concentration of radiolabeled glucose and hence tissue glycolysis (26). We found SUVmax and SUVpeak (30), which has been proposed as more accurate in small VOI and less influenced by a few voxels with high activity (31), to be useful, together with SUVmean which mitigated for the lack of a baseline scan by comparing injected and uninjected leg muscle. No increased ^18^F-FDG or ^11^C-PBR28 uptake was detectable after saline injection at any time point (**Figure 1E**). An intramuscular inclusion extending up to ~45 mm in the cranial - caudal axis and often branching was visible on CT scan at the site of vaccine injection at 3 hours (**Figure 2**), but not thereafter. A distinct VOI with increased uptake could be detected at the site of vaccine injection in both ^18^F-FDG- and ^11^C-PBR28-PET (**Figure 1**). The ^18^F-FDG PET/CT SUVmean and SUVmax of the immunization site VOI were increased relative to the contralateral uninjected or saline injected muscle at all time points post-immunization. The kinetics of the muscle PET activity are shown in **Figure 3**. A similar level and pattern of response kinetics for all measured parameters was evident after ATIV and STIV immunization, with a progressive increase in SUVmax and SUVpeak to day 3 before falling to near the 3 h level by days 7–10 (**Figure 3**). In contrast 4CMenB induced a higher and more prolonged increase with a maximal SUVmax and SUVpeak on days 3–5 followed by only a slight decrease up to day 10 but without returning to 3 hour levels, possibly due to a depot effect of the alum formulation. The kinetics and relative levels of activity of each vaccine were also similar when attenuation corrected but not weight standardised MBq/ml values were plotted, and even with uncorrected raw counts per millisecond (CPMS) from the PET, although a relationship with VOI volume could be seen with these parameters compared with SUV (**Figures 3C–F**) as is to be expected (26). We were able to scan two participants, using a different ^18^F-FDG PET/CT scanner, both on day 3 after immunization with AHBVV who both had a typical VOI of increased glycolysis detectable at the site of immunization with SUVmax, SUVpeak and VOI volumes of 2.3, 3.0 SUV; 1.8, 2.3 SUV; and 13.4, 36.7 cm^3^ respectively (data not shown on **Figure 3**).

**Figure 4:**
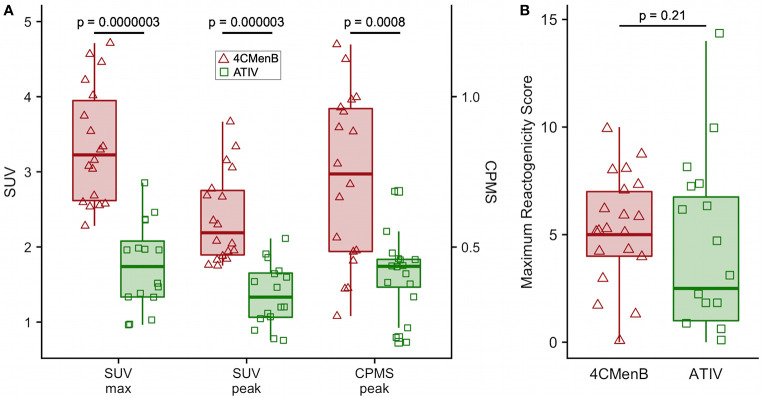
Comparison of 4CMenB and ATIV ^18^F-FDG PET/CT muscle activity and solicited symptoms severity scores. **(A)** SUVmax, SUVpeak and CPMS peak activity in muscle for all participants at all time points after receiving 4CMenB (pink, triangles) or ATIV (green, squares). **(B)** Maximum reactogenicity score on any day after immunization for all participants after receiving 4CMenB (red, triangles) or ATIV (green, squares). Horizontal bar: vaccine group median, shaded box: 95% confidence interval, whiskers: range. p values: Wilcoxon signed-rank test between vaccine groups.

Although this was a small preliminary study some exploratory statistical comparisons were made between injection site ^18^F-FDG PET/CT glycolysis in 4CMenB and ATIV immunized participants as comparable numbers and time points were available for these vaccines. The difference in the glycolysis recorded at the injection site was significantly higher after 4CMenB immunization whether SUVmax, SUVpeak or raw CPMS were compared (**Figure 4A**), which correlated with a non-significant trend to increased reactogenicity from diary card recording in participants immunized with 4CMenB (**Figure 4B**). The ^18^F-FDG PET/CT findings reflect the post licensing clinical experience as while 4CMenB has an acceptable safety profile (32), fever was recorded in 69–79% of infants co-administered with routine vaccines, for which prophylactic use of antipyretic medication has been recommended (33) in various countries; and in adolescents and adults pain at injection site, malaise, myalgia, arthralgia and headache are very common (34). *Neisseria meningitidis* OMVs contain immunostimulatory membrane components such as lipids, proteins and lipopolysaccharide (LPS) and while detergent extraction removes the majority of LPS, residual soluble endotoxin toxicity is ameliorated by complexing OMVs with alum (35). Nevertheless, *ex vivo* whole blood stimulation assays demonstrate that 4CMenB OMVs induced the release of inflammatory cytokines TNF and IL-1β, while alum appeared to have the effect of lowering IFNγ and chemokine CXCL10 (interferon inducible 10) release (36).

For ^11^C-PBR28-PET only 4CMenB immunization and scanning on days 1, 3, 5, and 7 were studied as the intention was only to determine whether PET/CT was sensitive enough to detect changes in ^11^C-PBR28 binding in immunized muscle and lymph nodes and experience with ^18^F-FDG PET/CT suggested this would be a good positive control. A distinct intramuscular VOI of increased binding was observed (**Figure 1E**) and increased SUV mean and SUV max compared with saline injected contralateral leg were detected in all subjects (**Figures 3G, H**). Generally the ^11^C-PBR28-PET kinetics were similar to those seen with ^18^F-FDG PET/CT at the same time points (**Figures 3G–J**).

### ^18^F-FDG and ^11^C-PBR28 PET Activity in Draining Lymph Nodes

**Figure 5:**
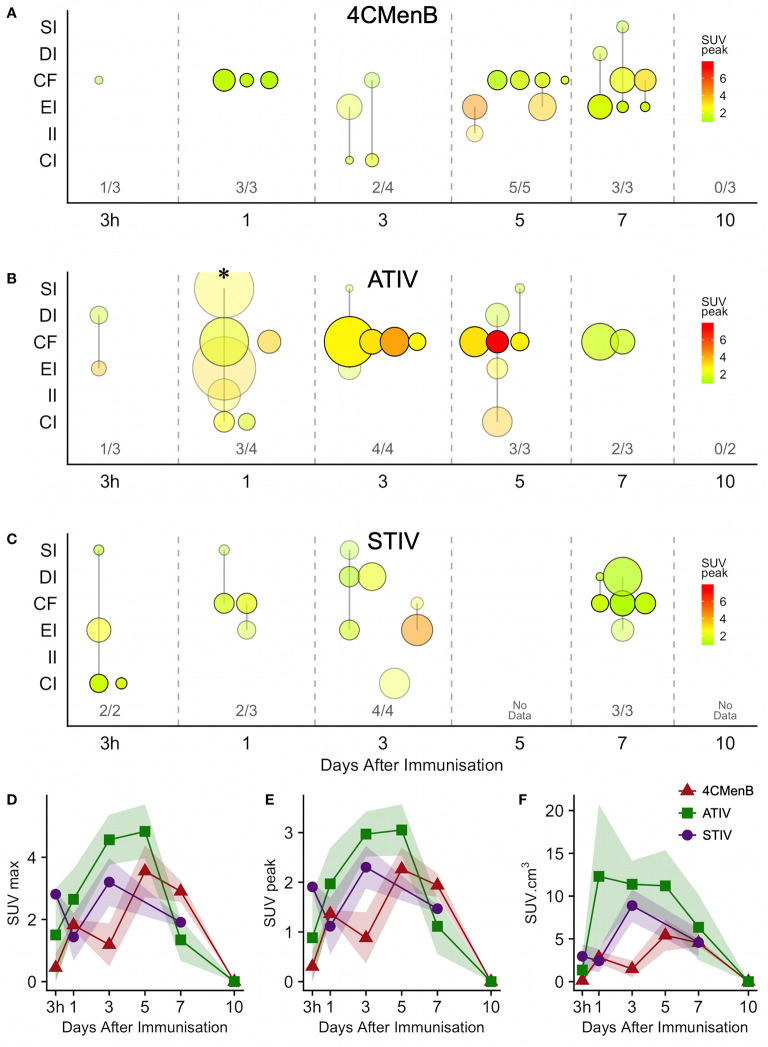
Anatomical distribution and kinetics of lymph node ^18^F-FDG PET activity after immunization. **(A–C)** bubble chart of lymph node ^18^F-FDG PET activity after immunization with 4CMenB **(A)**, ATIV **(B)** or STIV **(C)**. X axis: time between scan and immunization (3 h, 1 - 10 days). Y axis: anatomical lymph node group: SI - superficial inguinal; DI - deep inguinal; CF - common femoral; EI - external iliac; II - internal iliac; CI - common iliac. Bubble diameter: lymph node volume; bubble transparency: proportion of participants with detectable lymph node activity in each anatomical group per time point; bubble color: SUVpeak. Figures above X axis: number of participants with detectable lymph node activity at each time point per total participants in group. **(D–F)** Group mean SUVmax **(D)**, SUVpeak **(E)** and total lymph node glycolysis (SUVmean x volume), **(F)** at different time points after immunization with 4CMenB (red triangles), ATIV (green squares) or STIV (purple circles). Shaded area: SEM. * participant with widely disseminated lymph node activity (**Figure 1D**).

Increased glycolysis could be identified in at least one lymph node in 38/53 subjects studied with ^18^F-FDG PET/CT at some time after immunization (**Figures 5A–C**), which was ipsilateral to the side of vaccine injection in 31, contralateral in 3, and bilateral in 4 participants. As radiation exposure restrictions precluded a baseline scan it is not possible to exclude chance pre-existing increased lymph node glycolysis, although the strong ipsilateral association with vaccine injection and the kinetics of the lymph node SUV max, SUV peak and total lesion glycolysis (37) together with proportion of participants with detectable lymph nodes which showed an increase to a peak around days 3, 5, and 7 followed by a fall by day 10 (**Figures 5D, E**), strongly suggest the activation observed was induced by immunization. The anatomical location of lymph nodes (**Figures 5A–C**) also suggests a response to immunization as across all the vaccines the most frequently observed lymph node group was the common femoral (in 28 participants), which is to be expected as it is part of the deep inguinal lymph nodes which drain the thigh muscles; followed by the external iliac nodes which drain the deep inguinal nodes (15 participants), and then common iliac (8 participants), superficial inguinal and deep inguinal (7 participants each), and internal iliac (2 participants). Due to radiation exposure restrictions the attenuation CT scan was limited to the pelvis and thigh, which precluded accurate identification of extra-pelvic nodes, but within the pelvis 18/38 participants with identifiable lymph node activity had more than one lymph node group involved, but only one participant had widely disseminated lymph node activity–on day 1 after ATIV immunization (**Figure 1D** and indicated on **Figure 5B** by *). No participants scanned on day 10 had detectable lymph nodes, although data was not available for STIV. Increased lymph node PBR28 binding was also clearly visible with ^11^C-PBR28-PET (**Figure 1F**), which also labeled bone marrow as would be expected.

Generally ATIV immunization resulted in the highest lymph node volume and peak SUV (**Figure 5B**), which peaked on days 3 and 5 when all participants had detectable lymph nodes. This correlates with murine models in which intramuscular neutrophils and monocytes recruited by MF59C efficiently take up antigen, aided by the emulsion, and transport it to draining LNs (38) where the differentiation of monocytes to dendritic cells is enhanced by MF59C (39), resulting in enhanced triggering of antigen-specific CD4 T cell responses (39). Additionally, MF59C B cell adjuvanticity appears to be mediated by potent induction of T follicular helper cells which directly control LN germinal centre responses (40) and also correlate with long-term functional immunity (41). In contrast, we observed relatively smaller nodes with lower peak SUV (**Figure 5A**) after 4CMenB which was in marked contrast to the relative intensity of the associated muscle activation, and in keeping with murine models of immunization with OMVs containing native or genetically attenuated LPS which demonstrated that increased intramuscular inflammation was not associated with increased immunogenicity (36).

### Whole Blood and Muscle Biopsy Transcriptomics Analysis

Bergström needle muscle biopsies have been used for decades with a good safety record [minor complication rate of 0.15% (25)] and provide adequate samples for histologic, ultrastructural, DNA, RNA and enzyme analysis, from a wide range of muscles (25), although the larger mass of the anterior thigh muscles is most amenable, and is an acceptable site of immunization in UK (42). Adequate RNA for analysis was obtained from all whole blood samples, and all muscle biopsies except for one taken from the contralateral unimmunized leg on day 1 after ATIV immunization.

**Figure 6:**
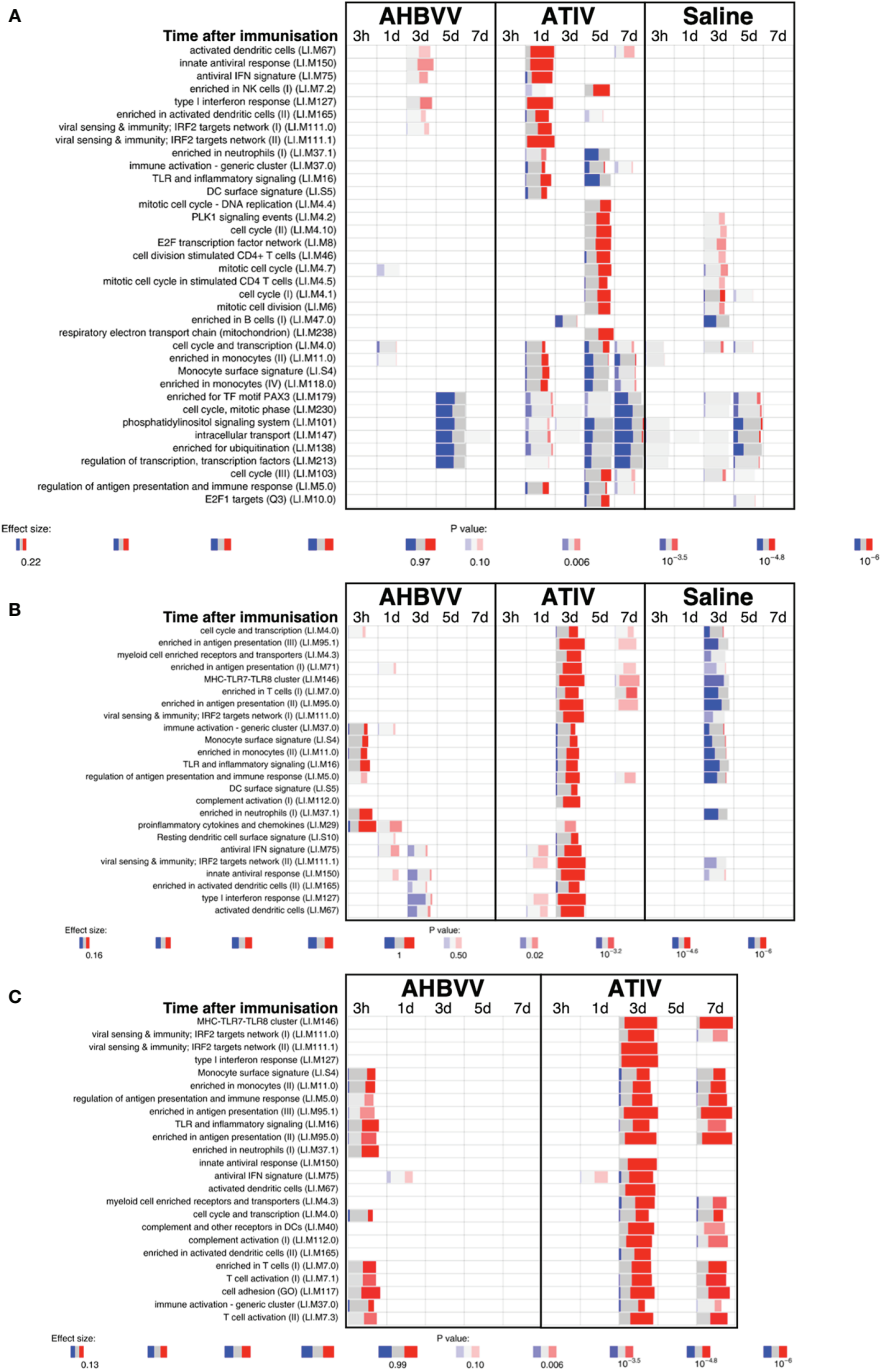
Gene expression in whole blood and muscle at different time points after immunization. Gene set enrichment analysis of signatures at different time points after immunization in the whole blood [pre-immunization time point control **(A)**] and immunized leg muscle biopsy [**(B)** paired unimmunized leg biopsy as control; **(C)** saline group as control]. Bar sizes correspond to effect size (AUC) in the enrichment and intensity of color to the adjusted p value of enrichment (stronger color corresponds to lower adjusted p value). Red and blue boxes indicate fractions of genes that have, respectively, a significantly higher or lower expression in the test group compared to control.

Analysis of whole blood gene expression in blood transcriptional modules (BTMs) after ATIV immunization (**Figure 6A**) revealed the kinetics we had observed in a previous study with a larger sample size (23) with upregulation on day 1 of modules relating to innate immunity, inflammation, monocytes and IFN-related responses (LI.M67, LI.M150, LI.M75, LI.M127, LI.M165, LI.M111.1, LI.M37.0, LI.M.16, LI.M11.0, LI.S4) and cell cycle/transcription (LI.M4.0) that were also seen in the previous study, plus additional related modules (LI.M111.0, LI.M37.0, LI.S5 and LI.M.118), and in LI.M5.0 (antigen presentation). As in the previous study no upregulated whole blood gene expression was seen on day 3, but upregulation of late-phase response modules governing CD4 T cells, C-MYC and PLK1 signalling (LI.M4.4, LI.M4.2, LI.M4.10, LI.M8, LI.M46, LI.M4.7, LI.M4.5, LI.M4.1, LI.M6, LI.M102, LI.M10.0) observed in the previous study were again seen on day 5, and correlate with the enhanced antigen-specific CD4+ T cell responses we previously observed with MF59C adjuvant using flow cytometry (41).

The kinetics of whole blood gene expression changes suggest a trafficking of immune cells to and from the site of immunization, and indeed analysis of ATIV immunized muscle biopsy gene expression (compared with the uninjected muscle–**Figure 6C**) revealed a strong upregulation of modules on day 3 that were also upregulated in whole blood on day 1 (LI.M4.0, LI.M111.0, LI.M37.0, LI.MS4, LI.M11.0, LI.M16, LI.M5.0, LI.MS5, LI.M75, LI.M111.1, LI.M150, LI.M165, LI.M127, LI.M67) and associated with antigen presentation and interferon responses; together with additional modules not seen in whole blood responses (LI.M95.1, LI.M4.3, LI.M71, LI.M146, LI.M7.0, LI.M95.0, LI.M112.0, LI.M29, LI.MS10) but also strongly associated with antigen presentation. Some of the modules upregulated in muscle on day 3 were also slightly upregulated in muscle on days 1 and 7. The kinetics of these gene expression changes correlated with the level of glycolysis seen in muscle detected by ^18^F-FDG PET/CT (**Figures 3A–F**), and correspond closely to murine models (38, 43) in which sequential muscle infiltration with neutrophils (peak 16 h), eosinophils/monocytes (peak days 2–3), and macrophages/dendritic cells (peak day 3) occurs, associated with chemokine MCP-1 (CCL-2), IL-8 (CXCL-8), CCL3 and CCL4 production. MF59C, unlike alum, also induces the release of extracellular ATP from muscle that may act as endogenous danger signal (44).

**Figure 7:**
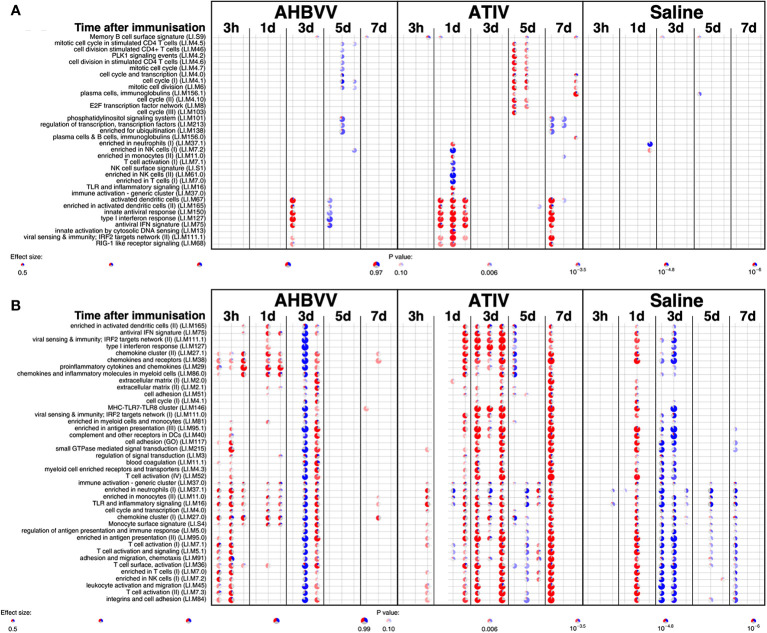
Gene expression in whole blood and muscle from individual participants at different time points after immunization. Gene set enrichment analysis of signatures at different time points after immunization in the whole blood [pre-immunization time point control **(A)**] and immunized leg muscle biopsy [paired unimmunized leg biopsy as control, **(B)**]. Pie diameters correspond to effect size (AUC) in the enrichment and intensity of color to p value of enrichment (stronger color indicates lower p value). Red and blue sectors indicate fractions of genes that have, respectively, a higher or lower [abs(log2FC) > 0.5] expression in test group compared to control.

MPL, present in AS04 (45) is a detoxified lipopolysaccharide (LPS) that signals through Toll-like receptor 4 (TLR-4) although in a different way to LPS (45). Alum does not appear to synergise with MPL but prolongs the cytokine responses over the first 24h *via* a depot effect (44). In contrast with ATIV, we observed that gene expression changes in whole blood were largely absent after immunization with AHBVV, apart from a signal on day 3 in one participant in a subset of BTMs also upregulated on day 3 after immunization with ATIV (**Figure 7A**). Also in contrast with ATIV, significant upregulated gene expression could be detected in the muscle biopsy from the site of immunization at 3 h after immunization with AHBVV (**Figure 6B**), in a subset of BTMs also upregulated in muscle on day 3 after ATIV immunization and associated with TLR signalling, monocytes, neutrophils and immune activation. Looking at the individual gene expression profiles of participants biopsied at 3 h, 1 and 3 days after AHBVV immunization (**Figure 7B**), 6/9 had upregulation of modules also upregulated in muscle on day 3 after ATIV immunization, albeit at lower levels; whereas none of the 6 biopsied on days 5 and 7 had significant upregulation of gene expression. The upregulation of BTMs we observed closely match the kinetics and phenotype of cellular infiltrates seen murine models in which AS04 induced intramuscular production of proinflammatory cytokines and chemokines within 3–6 h that returned to baseline by 22h (46), with an increase in activated antigen-loaded monocytes and dendritic cells within the first day after injection (44).

When gene expression of BTMs in the muscle biopsies from the site of immunization taken from participants immunized with AHBVV or ATIV was contrasted with that in the biopsies from the site of saline injection taken from participants in the control group (**Figure 6C**), the difference in the timing of the gene activation events between ATIV and AHBVV seen in the comparison between immunized and unimmunised leg biopsies was reinforced with upregulated gene expression in muscle on days 3 and to a lesser degree day 7 after immunization with ATIV, but at 3 h after immunization with AHBVV and involving a subset of the same BTMs seen after ATIV (**Figure 6C**).

We used lidocaine local anesthetic without epinephrine which has been shown to affect gene transcription (47) and took great efforts to accurately biopsy the same region of muscle that had been injected, by marking the injection site and engraving the biopsy needle to align the side port through which muscle is extracted with the exact length of the needle for injection. Nevertheless we observed significant inter-subject variation in the muscle gene expression (**Figure 7B**), which probably represents a mixture of technical biopsy issues together with biological variations in vaccine responses, which we also observed in whole blood gene expression in a previously published study (23). Furthermore, ^18^F-FDG PET/CT revealed that it might have been better to biopsy some millimeters deeper than the needle length where the peak activity was focused (**Figures 1A, E**), and as the injectate often took a bifurcating path (**Figure 2**) it is inevitable that this and operator technique meant we probably missed the muscle region with maximal changes on some occasions.

## Discussion

The evaluation of vaccine reactogenicity and tolerability is traditionally undertaken during Phase 1/2 clinical trials in which participants keep a diary card of solicited and unsolicited local injection site and systemic symptoms. However, the methodology is not precise as the symptoms are typically quantified only on a four level categorical scale based on interference with activities of daily living and tend to be skewed heavily to the first and occasionally second level (mild or moderate). This, together with inter-subject variation in perception of severity requires a large number of respondents to obtain meaningful data. While this technique may be satisfactory for the rule-out of unacceptable reactogenicity based on a benefit-risk calculation for licensing purposes, it is inefficient for the comparison of vaccine candidates and adjuvants, or fine-tuning vaccine formulations during development (1). The BIOVACSAFE project had as a major focus the application of systems vaccinology and high precision techniques to identify biomarkers of vaccine safety and reactogenicity. We therefore undertook an exploratory analysis of the ability of PET/CT scan to predict the known reactogenicity profile of these vaccines. In addition participants recorded symptoms in diary cards and we calculated a simple daily reactogenicity score based on the sum of the scores for each of the solicited symptoms. As may be expected from the published data on reactogenicity obtained during clinical development and post-licensure monitoring, the median of the maximum reactogenicity score recorded in diaries after 4CMenB immunization was higher than for ATIV, but because of the high inter-participant variance this was not clinically significant (**Figure 4B**), reinforcing the need for a large sample size when assessing vaccine reactogenicity this way. In contrast, the higher median SUVmax and SUVpeak detected by ^18^F-FDG PET/CT was highly significantly different (**Figure 4A** and **Table 1**), and therefore predicted the extended clinical experience with these vaccines using a very small number of participants.

We selected young adults as most representative of Phase 1/2 clinical vaccine trials, which required a 10 mSv total radiation dose limit (category IIb defined by the International Commission for Radiological Protection, effective dose 1 to 10 mSv). For comparison, the average annual natural background radiation dose in the UK is 2.7 mSv (6.9 in Cornwall) and 6.2 in the USA (48). We accommodated this ethical constraint by omitting a baseline PET/CT scan, to reduce the total radiation dose to 6.0 mSv (^18^F-FDG PET) or 3.9 mSv (^11^C-PBR28 PET) which corresponds to approximately 2.2/1.4 years natural background radiation in the UK, and 11.6/7.5 months in the USA for the ^18^F-FDG PET/^11^C-PBR28 PET protocols respectively. The risk from exposure to ionizing radiation is the induction of cancers and using the risk factor for a UK population of both sexes for ages 18−64 years has been estimated at 5% per Sievert (49), the lifetime risk of inducing a cancer in a healthy individual is therefore approximately 1 in 3300 from a dose of 6.0 mSv, although no epidemiological data actually exists for an increased risk from doses less than 10 mSv (50). This should be compared with the natural incidence rate for cancer in the UK of ~1 in 4. Furthermore, ^18^F-FDG radioactivity is usually eliminated from the body in a few hours, and as the half-life of ^11^C is 20 min most of the radioactivity is eliminated from the body before the subject leaves the imaging unit. While the intramuscular activity could be controlled by the contralateral unimmunized leg, we could not rule out baseline increased nodal activity, although the kinetics and distribution of observed nodal activation strongly suggests a response to the vaccines. We also chose to do a CT scan to allow PET activity correction for tissue attenuation and the anatomical location of increased activity—especially within lymph nodes. However, it is interesting that the intramuscular ^18^F-FDG PET activity measured only by the CPMS, without correction for tissue attenuation or the body mass of the participant, was still highly significantly different between 4CMenB and ATIV (**Figure 4A**), suggesting that PET alone could be used to quantify the early intramuscular inflammatory immune events, and PET may be calibrated using fixed radiation sources. Therefore, the total radiation dose could be reduced by omitting the CT scan which could permit baseline and serial scans on the same participant. Alternatively, PET/MRI scanning is increasingly available and has been shown to work with tissue-specific radioligands such as ^11^C-PBR28 (51). These alternatives, together with potentially injecting different formulations and controls into the limbs of the same participant, or recruiting older participants for whom a higher total dose is acceptable, would allow the quantification and kinetics of the inflammatory events that will lead to the immune responses to different vaccines, adjuvants, formulations or doses to be evaluated in far fewer people than are currently required in Phase 1 and 2 trials.

While the application of systems biology techniques to biopsy material is an attractive way to study immune interactions following human immunization, it would be preferable to employ non-invasive techniques where possible. The development of highly specific radioligands suitable for clinical use that can be radiolabeled and target defined tissue or cell markers is now well established in the study of neurological disease, malignancy and pharmacokinetics. However, the use of such radioligands to characterize immune activation events following immunization has not been reported. We therefore took advantage of local expertise with one such ligand, PBR28 and the availability of an onsite cyclotron and scanning facility to conduct a very preliminary study of ^11^C-PBR28-PET/CT responses to immunization, selecting 4CMenB and days 1–7 scans to optimize the likelihood of detecting a signal. The 18-kDa translocator protein (TSPO; formerly known as the peripheral benzodiazepine receptor, PBR) is a mitochondrial membrane protein involved in steroidogenesis and cholesterol transport expressed throughout the body, and highly upregulated in microglia, macrophages (52) and CD4+ T cells (53) during inflammation. It has therefore been used as a PET imaging target for the investigation of inflammatory diseases involving microglial activation and/or macrophage recruitment (54–58). As the affinity of PBR28 depends on a single polymorphism (rs6971) in the TSPO gene (59), individuals who expressed only low-affinity TSPO were excluded by genetic screening. We postulated that ^11^C-PBR28 would identify activated macrophages and other immune cells at the vaccine injection site and draining lymph nodes. Indeed, ^11^C-PBR28-PET/CT revealed a well-defined VOI with increased ^11^C-PBR28 binding at the site of immunization in all participants scanned between days 1–7 (**Figures 1** and **3**), which correlated well with ^18^F-FDG PET/CT, and further supports the expected infiltration of immune cells. Increased ^11^C-PBR28 binding within draining lymph nodes was also evident, with sharply defined anatomy in comparison with ^18^F-FDG/CT (**Figure 1**). These findings suggest that PET/CT employing radiolabeled molecules such as monoclonal antibodies certified for clinical use targeting immune cell surface markers or cytokines and chemokines within target tissues may be a practical technique to characterize *in vivo* immune responses to vaccines, and to quantify pro-inflammatory and pro-immunogenicity responses to different adjuvants, formulations and doses in humans.

While whole blood gene expression is an effective way to study trafficking immune cells, there is increased interest in applying systems biology and high precision techniques to sites of immune interaction in humans, especially lymph nodes (60). In this study we complemented our previously published (23) studies comparing whole blood gene expression responses of humans to different live, subunit and adjuvanted/unadjuvanted vaccines, to further characterize the muscle activation observed on PET/CT by obtaining tissue biopsies from the injection site after intramuscular immunization with the adjuvanted vaccines AHBVV and ATIV, or a saline control. The kinetics and gene signatures associated with activated BTMs correlated very well with animal models characterizing the cellular infiltrates and immune responses at the site of immunization and in draining lymph nodes, and “closed the loop” on the patterns previously seen in whole blood by demonstrating the tissue migration and then efflux of activated cells (summarised in **Table 1**). This study demonstrates that tissue biopsy after immunization is both practical and ethical in human models of immunity and inflammation. While the Bergström technique is safe, well tolerated and provides a large tissue sample (25), it may be that multiple, radiologically-guided fine needle aspirates may be more efficient at obtaining a representative muscle biopsy with less trauma. Our PET/CT data also show that different vaccines and adjuvants induce different patterns of lymph node activation, and while the anatomical location of activated lymph nodes may be broadly predicted by knowledge of drainage patterns, there was significant inter-participant variation with often only one activated node in a group. ^18^F-FDG PET/CT, combined with guided fine needle aspiration therefore offers a technique to increase the chance that only lymph nodes that are actually responding to immunization are aspirated and at the optimal time point, which is especially important in systems biology and high precision techniques where practical sample sizes are often small and aggregated data may be biased by non-responding tissue samples. Indeed in a recent human study of immunization with an unadjuvanted influenza vaccine (60), weekly lymph node aspirates provided useful samples for flow cytometry in only 3/8 participants, which the authors concluded could have been due to the aspiration of a non-responding node and/or mis-timing the response. In our studies with 4CMenB, ATIV and STIV we observed a peak response in lymph nodes around days 3–5, which contrasts with some case series where increased lymph node ^18^F-FDG PET/CT activity could be detected some weeks after immunization. However, these studies were opportunistic retrospective observations in patients undergoing ^18^F-FDG PET/CT as part of cancer or other treatment, and in whom the exact vaccine and immunization date was not recorded as part of the study, and highlights the importance of prospectively and systematically determining the kinetics of ^18^F-FDG PET/CT and tissue site responses for each vaccine candidate or adjuvant to optimize systems vaccinology and high precision imaging techniques.

In conclusion we have demonstrated that PET/CT and gene expression analysis of tissue biopsies are ethically and practically acceptable for intensive, small scale predictive clinical studies of vaccine inflammatory and immune responses, and with the application of target-specific radioligands, radioactive dose-reducing techniques and temporally- and anatomically-guided biopsies may contribute to a better understanding of the balance between inflammatory and immune responses, and the selection and optimisation of candidate vaccines and adjuvants with shorter development times and enhanced safety and reactogenicity profiles.

## Data Availability Statement

The datasets presented in this study can be found in online repositories. The name of the repository and accession number can be found here: https://www.ncbi.nlm.nih.gov/geo/, GSE124719.

## Ethics Statement

The studies involving human participants were reviewed and approved by London - Surrey Borders Research Ethics Committee Health Research Authority. The participants provided their written informed consent to participate in this study.

## Author Contributions

ZW interpretation and analysis of PET/CT, manuscript review. JW analysis of transcriptomics, manuscript review. NP and RS design of PET/CT protocols, conducting scans, manuscript review. JM, H-JM, and KP preparation and processing of tissue samples, study design, manuscript review. AG and AL collection of samples, clinical management, manuscript review. TC and CB clinical project management, manuscript review. PD, GG, and SK manuscript review. DL project design, manuscript initial draft, and review. All authors contributed to the article and approved the submitted version.

## Funding

This is a summary of independent research carried out at the NICRF and funded in part by the Imperial Biological Research Centre. The views expressed are those of the authors and not necessarily those of the funders, NHS, the NIHR or the Department of Health. Funding was also received from the Innovative Medicines Initiative Joint Undertaking “BIOVACSAFE” under grant agreement no. 115308, resources of which are composed of financial contribution from the European Union’s Seventh Framework Programme (FP7/2007-2013) and EFPIA companies’ in kind contribution; and the European Union’s Seventh Framework Programme under grant agreement no. 280873 “ADITEC”.

## Conflict of Interest

PD and GG are employees of the GSK group of companies, and report receiving restricted shares of the company.

The remaining authors declare that the research was conducted in the absence of any commercial or financial relationships that could be construed as a potential conflict of interest.
